# Caveolin-1-mediated STAT3 activation determines electrotaxis of human lung cancer cells

**DOI:** 10.18632/oncotarget.21306

**Published:** 2017-09-28

**Authors:** Li Li, Kejun Zhang, Conghua Lu, Qin Sun, Sanjun Zhao, Lin Jiao, Rui Han, Caiyu Lin, Jianxin Jiang, Min Zhao, Yong He

**Affiliations:** ^1^ Department of Respiratory Disease, Daping Hospital, Third Military Medical University, Chongqing 400042, China; ^2^ Department of Clinical Laboratory, Daping Hospital, Third Military Medical University, Chongqing 400042, China; ^3^ School of Life Sciences, Yunnan Normal University, Kunming 650500, China; ^4^ State Key Laboratory of Trauma, Burns and Combined Injury, Daping Hospital, Third Military Medical University, Chongqing 400042, China; ^5^ Department of Dermatology, Institute for Regenerative Cures, University of California, Davis, CA 95817, USA

**Keywords:** electrotaxis, lung cancer, directional migration, caveolin-1, STAT3

## Abstract

Migration of cancer cells leads to the invasion of distant organs by primary tumors. Further, endogenous electric fields (EFs) in the tumor microenvironment direct the migration of lung cancer cells by a process referred to as electrotaxis – although the precise mechanism remains unclear. Caveolin-1 (Cav-1) is a multifunctional scaffolding protein that is associated with directional cell migration and lung cancer invasion; however, its precise role in lung cancer electrotaxis is unknown. In the present study, we first detected outward electric currents on the tumor body surface in lung cancer xenografts using a highly-sensitive vibrating probe. Next, we found that highly-metastatic H1650-M3 cells migrated directionally to the cathode. In addition, reversal of the EF polarity reversed the direction of migration. Mechanistically, EFs activated Cav-1 and the downstream signaling molecule STAT3. RNA interference of Cav-1 reduced directional cell migration, which was accompanied by dampened STAT3 activation. Furthermore, pharmacological inhibition of STAT3 significantly reduced the electrotactic response, while rescue of STAT3 activation in Cav-1 knock-down cells restored electrotaxis. Taken together, these results suggest that endogenous EFs in the tumor micro-environment might play an important role in lung cancer metastasis by guiding cell migration through a Cav-1/STAT3-mediated signaling pathway.

## INTRODUCTION

Cell migration plays a crucial role in many biological processes, including embryonic development, wound healing and immune surveillance, and cancer invasion [1, 2]. Migration of cancer cells leads to invasion of distant organs by primary tumors, which is the essence of tumor metastasis, accounting for more than 90 percent of all cancer related deaths [3, 4]. Metastasis is a multistep process where tumor cells disseminate from the primary tumor and colonize distant organs. During dissemination, cancer cells achieve directed cell migration through molecular coordination of a number of matrixed activities that are referred to as protrusion, chemotaxis, and contractility [5]. However, the biological and molecular mechanisms that are involved in the directed migration of tumor cells is poorly understood.

Directed cancer cell migration is controlled by various environmental factors, including the extracellular matrix (ECM), cytokines and growth factors, and their interacting cognate receptors that transduce chemotactic signals [5]. Aside from transduced chemical signaling cues, physical factors such as direct current electric fields (dcEFs) might also play a role [6, 7]. Physiological dcEFs were found to exist in living organisms in both plants to animals, and at sites of wound healing, regeneration and within tumors [8, 9]. In addition, dcEFs have been suggested to control wound healing, to polarize cells, and to guide directional cell migration - a process termed electro- or galvanotaxis. [7, 10].

Endogenous EFs could be an important migration cue for cancer cells [11], and guide the directional migration of several types of cancer cells including prostate, breast, and lung cancer cells [12–14]. Furthermore, the degree of electrotaxis of cancer cells correlates with their metastatic abilities. For example, in two sub-clones of human lung adenocarcinoma cells, the highly invasive CL 1-5 cells migrated directionally to the anode, while the low invasive CL 1-0 cells were non-electrotactic [15]. Similar phenomena were also observed in the electrotaxis of other cancer cells [13, 16]. However, it remains unclear why highly metastatic cancer cells display improved or superior electrotaxis activity.

Caveolin-1 (Cav-1) is an essential constituent protein of specialized membrane invaginations, which are referred to as caveolae [17]. Multiple cancer-associated processes are regulated by Cav-1, including cellular transformation, cell death and survival, multidrug resistance, and cell-mediated migration and metastasis [18]. In lung cancer, increased expression of Cav-1 was observed upon advanced progression of the disease [19]. In two cell-lines that were derived from metastatic lesions of lung cancer, Cav-1 was expressed at high levels, and siRNA-mediated knock-down (KD) of Cav-1 expression arrested cellular proliferation [20]. Cav-1 signaling has also been implicated in cancer invasion and directional cell migration. For example, stromal Cav-1 facilitates tumor invasion through force-dependent organization of the microenvironment [21]. Cav-1 was required for establishing polarization and externally stimulated directional migration of fibroblasts [22]. Given the important role of Cav-1 in cancer invasion and directional cell migration, and the phenomenon that cancer cells displaying an inherently higher invasive ability also respond better to an EF, we asked whether Cav-1 signaling participates in improved electrotaxis of highly-invasive lung cancer cells.

In the current study, we used two lung cancer cell-lines that were referred to as H1650 and H1650-M3, of which the invasive ability and drug sensitivity were detailed in our previous study [23]. Parental H1650 cells displayed low-metastatic potential, while H1650-M3 cells display high metastatic potential with enhanced activation of Cav-1. We quantitatively characterized electrotaxis of both cell-lines and the associated signaling events and found that H1650-M3 cells migrated far more directionally than H1650 cells in an EF. In addition, EF stimulation activated both Cav-1 and downstream STAT3, whereas knock-down of Cav-1 with shRNA or pharmacological inhibition of STAT3 activation decreased electrotaxis of H1650-M3 cells and STAT3 activation. Furthermore, rescue of STAT3 activation in Cav-1 knock-down cells restored directional cell migration. These results suggested that Cav-1-mediated STAT3 activation determines electrotaxis of highly-invasive lung cancer cells.

## RESULTS

### Presence of electric currents in the tumor microenvironment of lung cancer xenografts

**Figure 1:**
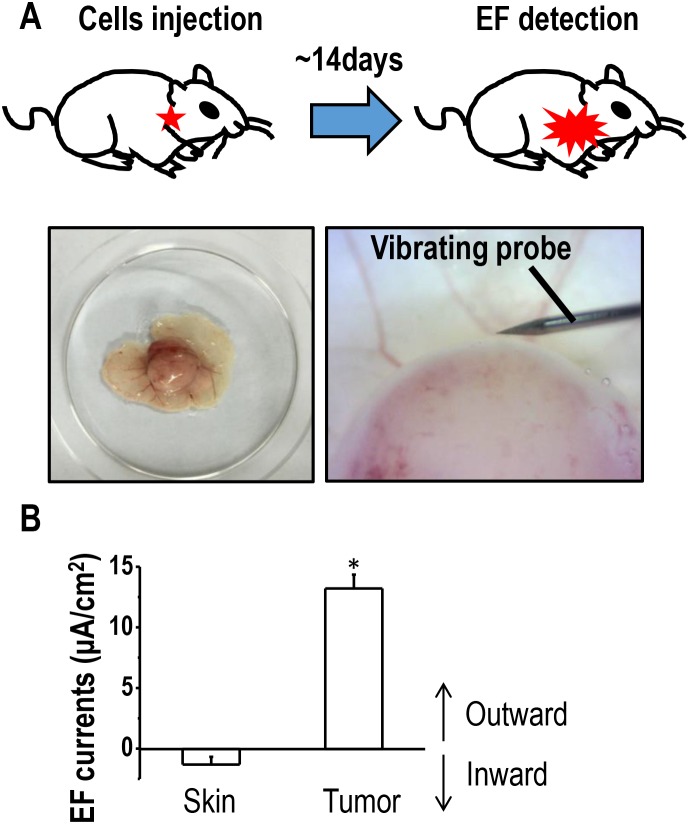
Outward electric currents exist at sites of lung tumors **(A)** Schematic figure of the xenograft model used in the current study. Full-thickness dermal specimens with the tumor was peeled off, placed into mouse ringer solution, following which, endogenous EFs around the tumor were detected using a vibrating probe. **(B)** Endogenous electric currents at tumor sites and normal uninvolved skin located nearby. The data are shown as mean ± S.E.M. ^*^, p<0.01 as compared with the control group. Similar results were obtained in three independent experiments.

Previous studies indicated that endogenous electric currents could be measured at skin wounds or in the cornea using a non-invasive vibrating probe [24, 25]. We first asked whether endogenous EFs existed around lung tumors. To investigate this, mouse xenografts were established based on lung cancer H1650-M3 cells, and the non-invasive vibrating probe technique was used to detect electric currents at the surface of a tumor (Figure 1A). Small inward currents were found at intact epithelium that were located approximately 3 cm away from the tumor body. Moreover, at the surface of the tumor, large outward currents were detected, which was statistically greater than that found at the intact epithelium around the tumor (Figure 1B). Such an outward current on the surface of a tumor implies the existence of an established EF between lung cancer and the surrounding tissues, with the anode being located inside the tumor and the cathode outside the tumor.

### Electric fields guide the directional migration of highly-metastatic H1650-M3 cells

**Figure 2:**
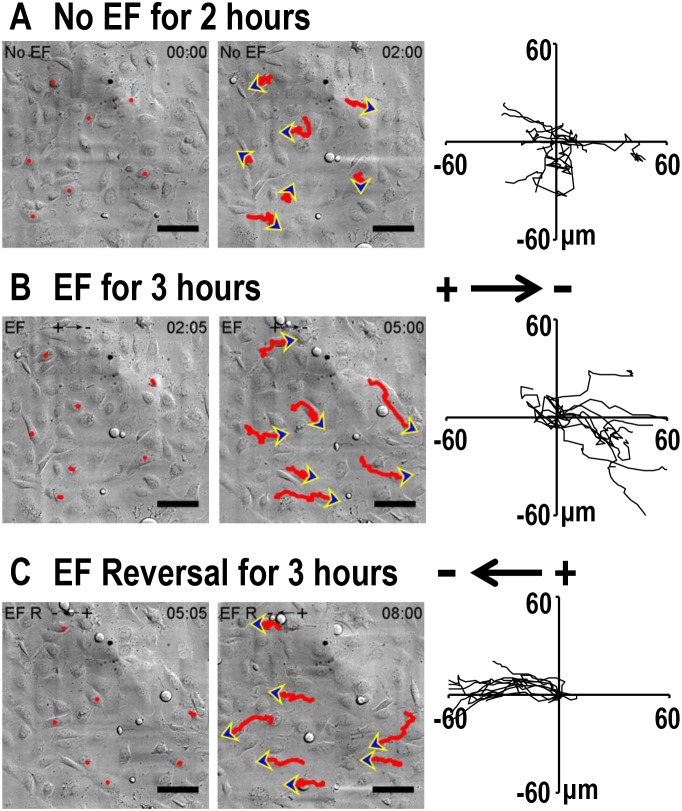
Electric fields guide directional migration of H1650-M3 cells to the cathode **(A)** Time lapse photographs of H1650-M3 cells in the absence of EFs for 2 hrs. Red lines and blue arrows represent migration paths and direction, respectively. Accumulated migration trajectories are presented with starting positions that were placed at the origin (0, 0). **(B)** H1650-M3 cells migrated directionally towards the cathode (i.e., to the right). EF = 100 mV/mm for 3 hrs. **(C)** After the EF polarity was reversed, cells of the same field continued to be recorded for an additional 3 hrs. H1650-M3 cells migrated to the new cathode upon reversal of the EF polarity. Scale bars = 50 μm. Also see Supplementary Movie 1.

**Figure 3:**
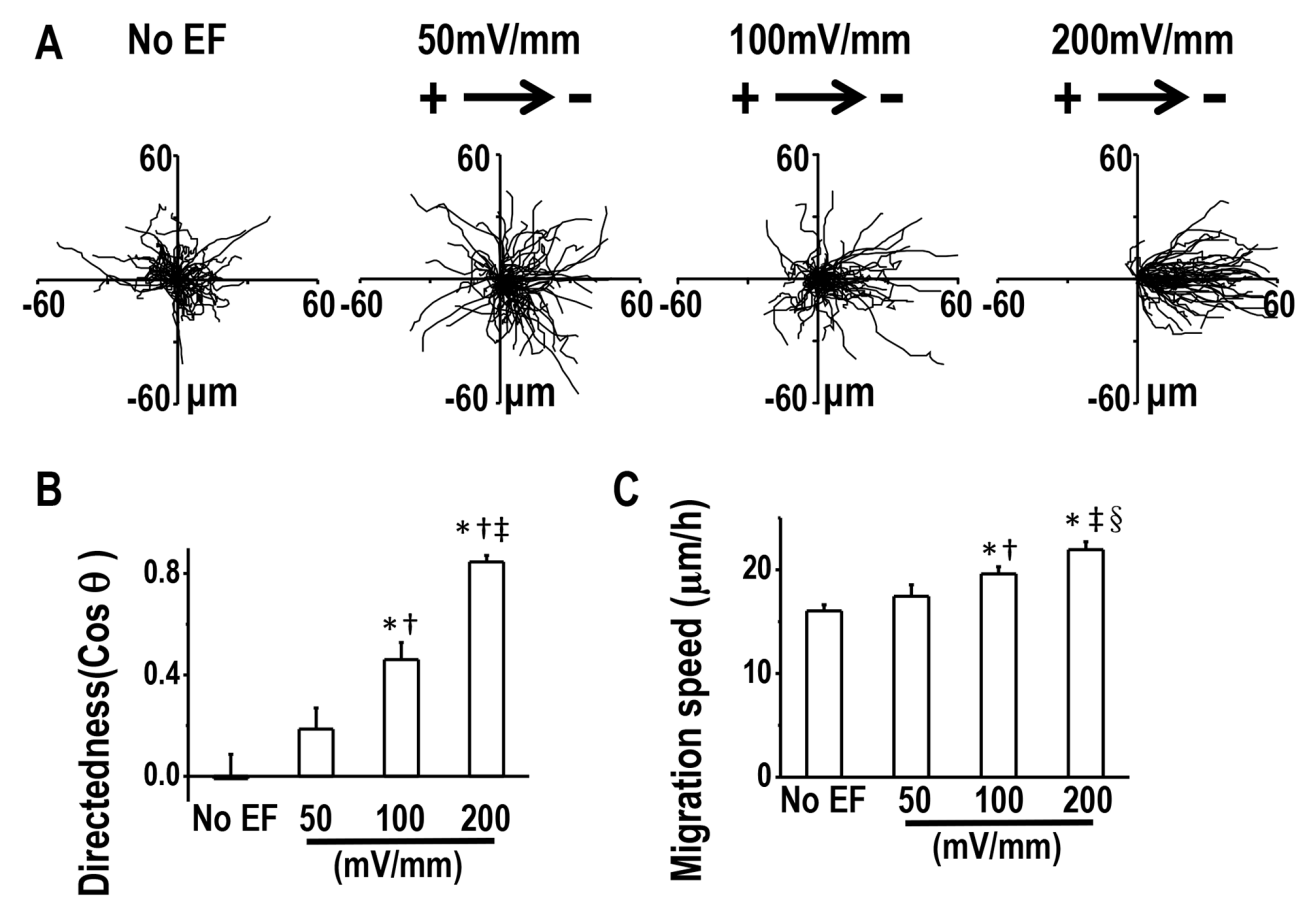
Directional migration of H1650-M3 cells in EFs are voltage-dependent **(A)** Migration tracks of H1650-M3 cells without EF stimulation or in EFs of different strengths for 2 hrs. Starting positions of cell migratory trajectories were placed at the origin. **(B)** Migratory directedness of H1650-M3 cells at different field strengths as indicated. ^*^, p<0.01 as compared with no EF control; †, p<0.01 as compared with that of 50 mV/mm; ‡, p<0.01 when compared with that of 100 mV/mm. **(C)** Migration speeds under different EF voltages. ^*^, p<0.01 as compared with no EF control; †, p<0.05 as compared with that of 50 mV/mm; ‡, p<0.01 when compared with that of 50 mV/mm; §, p<0.05 when compared with that of 100 mV/mm. Data are derived from at least 100 cells from 3 independent experiments and shown as mean ± S.E.M.

We next asked whether lung cancer cells of varying metastatic potential showed altered galvanotaxis in a small EF. H1650-M3 cells are highly metastatic human lung cancer cells, which were derived from parental low-metastatic H1650 cells. Cells were first left untreated for 2 hrs, and then exposed to an EF of 100 mV/mm for 3 hrs, which was then followed by a reversal of the EF polarity for an additional 3 hrs. In the absence of EFs, H1650-M3 cells migrated in all directions. By contrast, when the EF was turned on, cells migrated directionally to the cathode, as demonstrated by single cell tracking analysis (Figure 2A and 2B, also see Supplementary Movie 1). The electrotactic response of H1650-M3 cells was further evidenced by the observation that reversal of the EF polarity reversed the migratory direction. As shown in Figure 1C, cells migrated to the new cathode with displayed an even higher directional migration after the EF polarity was reversed. This phenomenon of “time-dependent electrotactic responses” has been reported before [10]. We then tested the voltage dependence of directional migration of H1650-M3 cells and found a gradually increasing cathodal migration when cells were stimulated with higher EFs (Figures 3A and 3B), while the migration speeds were also increased by stimulation with higher EFs (Figure 3C). Of note, despite the observation that most cells migrate directionally in an EF, a small proportion of the cell population did not respond well, with some even migrating in the opposite direction. For example, in an EF of 100 mV/mm, we found that approximately 10 percent of the cells migrated in the opposite direction. This might reflect heterogeneity of the cancer cell population. We next studied the electrotactic response of low-metastatic parental H1650 cells. In contrast to H1650-M3 cells, H1650 cells responded poorly to an EF of the same strength, and did not show directional migration (Supplementary Figure 1 and Supplementary Movie 2). Taken together, these results suggest that EF guides the directional migration of highly-metastatic lung cancer H1650-M3 cells in a voltage-dependent manner.

### EFs induced phosphorylation of Caveolin-1, which is required for electrotaxis

**Figure 4:**
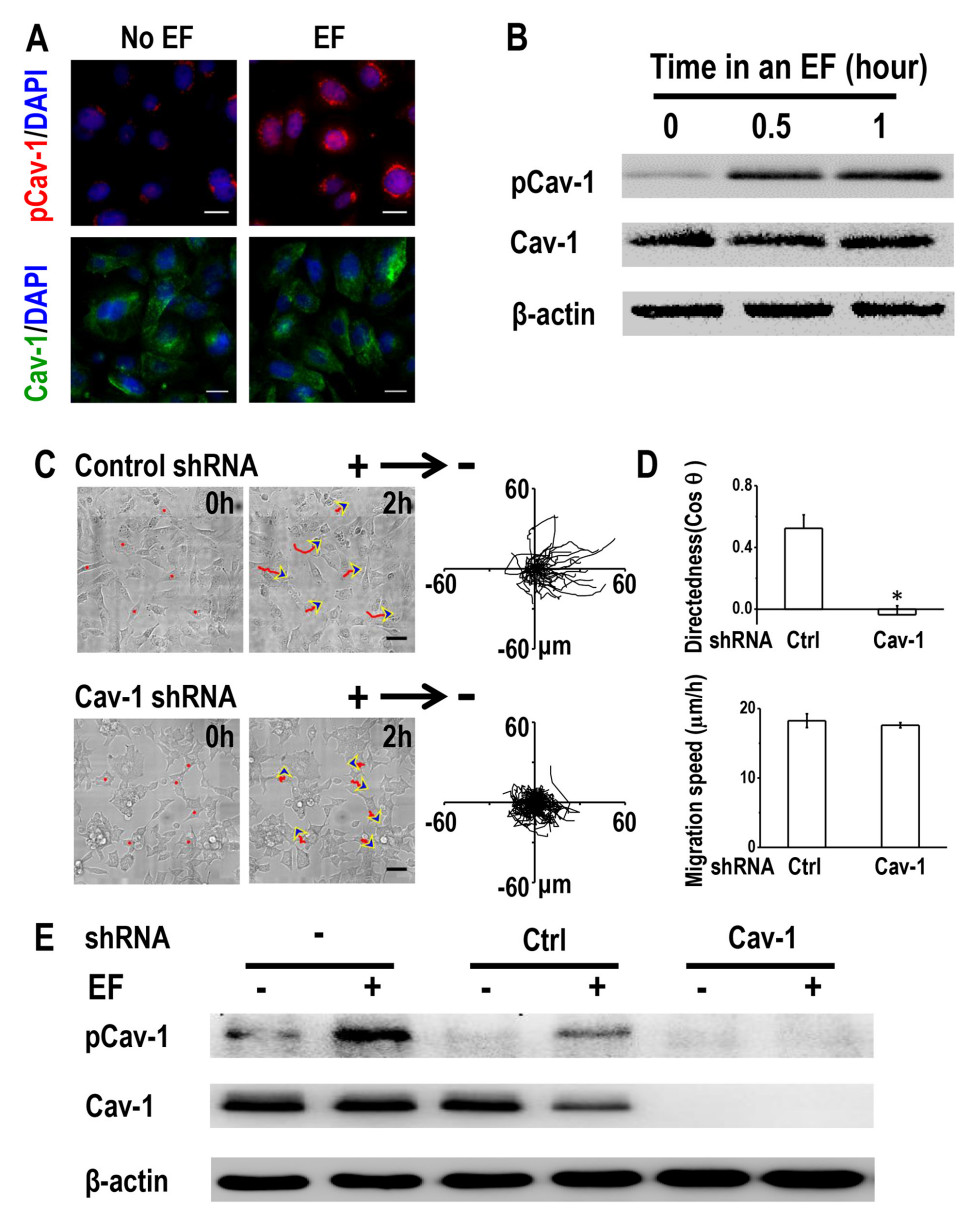
Caveolin-1 plays an essential role in electrotaxis of H1650-M3 cells **(A)** EF stimulation activates Caveolin-1 in H1650-M3 cells. Cells were left untreated or were exposed to an EF of 100 mV/mm for 2 hrs, fixed in paraformaldehyde and treated with anti-pCav1 (Tyr14) or anti-Cav1 antibodies, respectively. The nucleus was stained with 4’, 6-diamidino-2-phenylindole. Scale bars = 30 μm. **(B)** Whole cell protein lysates from H1650-M3 cells that were treated with an EF of different periods were immunoblotted with the indicated antibodies. Similar results were obtained in three independent experiments. EF = 100 mV/mm. **(C)** RNA interference of Caveolin-1 reduced electrotactic responses of H1650-M3 cells. H1650-M3 cells transfected with control shRNA or shRNA against Caveolin-1 were exposed to an EF of 100 mV/mm for 2 hrs. Red lines and blue arrows represent migration paths and direction. Scale bars = 50 μm. Cell migration trajectories are presented with the start point of each cell set at the origin. **(D)** Migratory directedness and speed of the indicated cells under an EF of 100 mV/mm for 2 hrs. The data are shown as mean ± S.E.M. ^*^, p<0.01 as compared with the control group. **(E)** Western Blot analysis was performed to detect the expression of pCav-1 and Cav-1 under different treatments as indicated in the text. Similar results were obtained in three independent experiments. EF = 100 mV/mm for 2 hrs. Cav-1: Caveolin-1. See Supplementary Movie 3.

Given the importance of Cav-1 in cancer invasion [20] and the observation that Cav-1 expression was much higher in H1650-M3 cells than was seen in H1650 cells (Supplementary Figure 2), we next investigated whether Cav-1 was involved in electrotaxis of lung cancer cells. An EF of 100 mV/mm for 2 hrs increased the phosphorylation of Cav-1 (Tyr14) in H1650-M3 cells, without markedly altering the expression of Cav-1 as shown by immunofluorescence staining (Figure 4A). Western blot results further demonstrated that the expression of phosphorylated Cav-1 (Tyr14) was enhanced by EF stimulation in a time-dependent manner, while the protein expression of total Cav-1 remained unaltered (Figure 4B). EF stimulation had little effect on the expression of phosphorylated Cav-1 (Supplementary Figure 3). These results suggested that Cav-1 was activated by EF stimulation in H1650-M3 cells.

We next investigated the requirement of Cav-1 in regulating H1650-M3 electrotaxis by short hairpin RNA (shRNA) knock-down of its expression. H1650-M3 cells were transfected with either control shRNA or vector containing shRNA targeting Cav-1. Western blot analysis confirmed that the expression of either phosphorylated or total Cav-1 was nearly abolished in Cav-1 knock-down cells (Figure 4E). When subjected to an EF, cells transfected with control shRNA remained as highly electrotactic as that of the parental H1650-M3 cells, with directional migration to the cathode. However, shRNA knock-down of Cav-1 triggered a significantly reduced electrotactic response (Figure 4C and 4D, Supplementary Movie 3). Concordantly, enhanced phosphorylation of Cav-1 under an EF was observed in both parental H1650-M3 cells and in cells with control shRNA, while knock-down of Cav-1 abolished EF-induced Cav-1 activation (Figure 4E). Taken together, our data indicated that activation of Cav-1 is required for electrotaxis of H1650-M3 cells.

### STAT3 activation is required for directional migration of H1650-M3 cells in an EF

**Figure 5:**
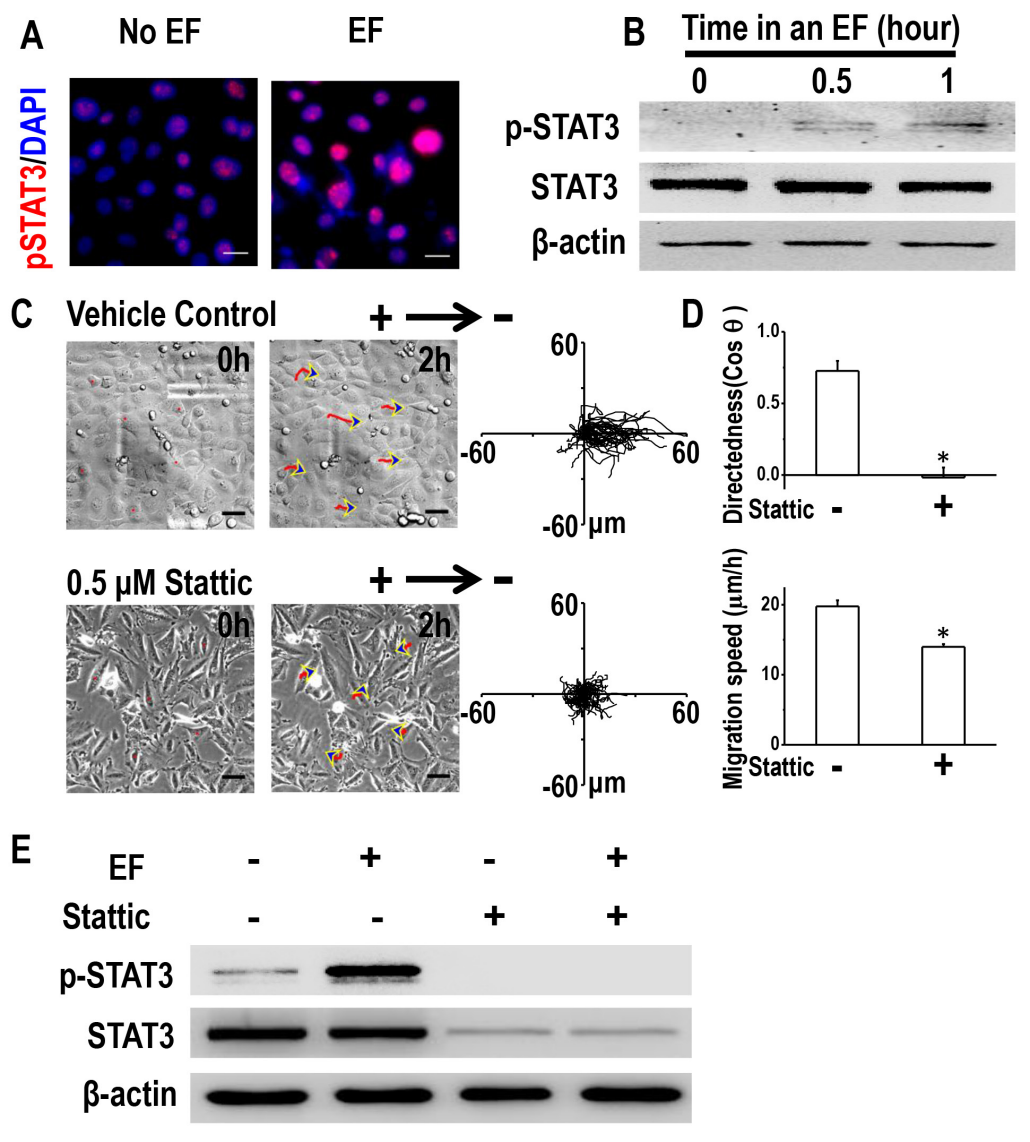
STAT3 activation is required for galvanotaxis of H1650-M3 cells **(A)** Immunofluorescence staining showed that expression of p-STAT3 was increased by EF stimulation (100 mV/mm for 2 hrs). The nucleus was stained with 4’, 6-diamidino-2-phenylindole in the merged images. Scale bars = 30 μm. **(B)** Whole cell protein lysates from H1650-M3 cells treated with an EF of different periods were immunoblotted with the indicated antibodies. Similar results were obtained in three independent experiments. EF = 100 mV/mm. **(C)** Pretreatment with Stattic (0.5 μM, 1 hr pretreatment and continuous presence) significantly inhibited directional responsiveness of cells to EF stimulation. Scale bars = 50 μm. EF = 100 mV/mm for 2 hrs. **(D)** Migration directedness and speeds of cells in an EF with/without Stattic. Data are from at least 100 cells from 3 independent experiments and shown as mean ± S.E.M. ^*^, p<0.01 as compared with the control group. **(E)** inhibition of STAT3 activation decreased the phosphorylation of STAT3 under an EF. Whole cell protein lysates from differentially treated H1650-M3 cells were immunoblotted with antibodies as indicated in the text, and β-actin was used to confirm equal gel loading. EF = 100 mV/mm for 2 hrs. Similar results were obtained in three independent experiments. See Supplementary Movie 4.

Knowing that STAT3 plays an important role in cancer invasion [26, 27], and that Cav-1 controls directional cell migration through STAT3 [20], we next asked whether STAT3 activation was required for EF-directed migration of H1650-M3 cells. Phosphorylated STAT3 was highly expressed in H1650-M3 cells when compared to H1650 cells; however, no significant difference was detected with regard to expression of total STAT3 (Supplementary Figure 2). Immunofluorescence staining showed that pSTAT3 in H1650-M3 cells was significantly enhanced after EF stimulation (Figure 5A). Western blot assay showed that EF activated STAT3 in a time-dependent manner (Figure 5B). To investigate whether STAT3 activation is required for H1650-M3 electrotaxis, we conducted electrotactic analysis in the absence or presence of the STAT3 specific inhibitor Stattic. Pharmacological inhibition of STAT3 triggered a profound reduction in both migration directedness and speed (Figure 5C and 5D, Supplementary Movie 4). Furthermore, inhibition of STAT3 activation abolished EF-induced activation of STAT3 (Figure 5E). The above observation suggested that STAT3 activation plays an essential role in the directional migration of H1650-M3 cells in an EF.

### Caveolin-1 determines electrotaxis of H1650-M3 cells by activating STAT3

**Figure 6:**
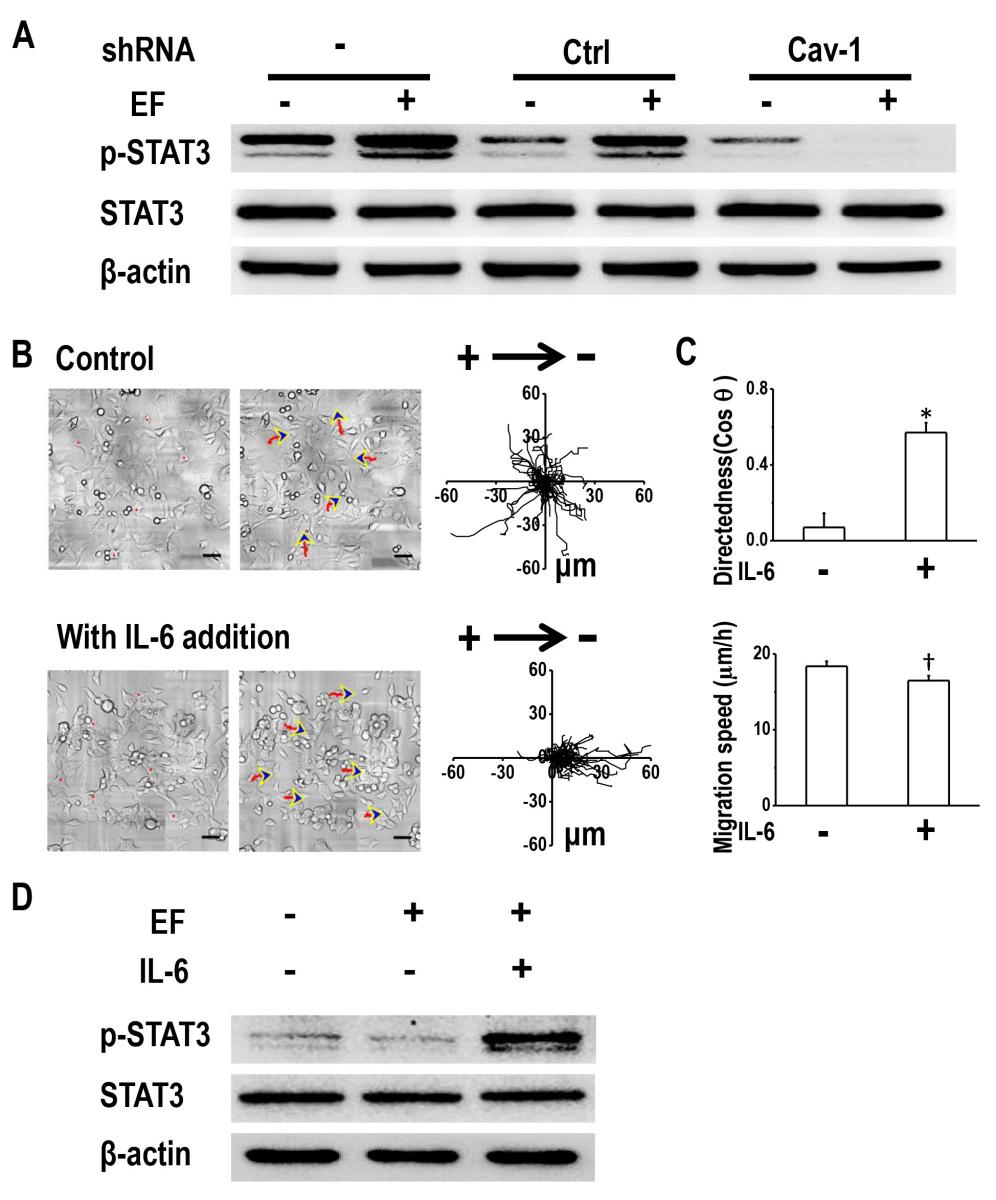
Cav-1-mediated STAT3 activation determines electrotaxis of H1650-M3 cells **(A)** Cav-1 KD decreased EF-induced activation of STAT3. Whole cell protein lysates from different cells with/without EF stimulation were immunoblotted with indicated antibodies. The expression of β-actin confirmed equal gel loading. Similar results were obtained from three independent experiments. EF = 100 mV/mm for 2 hrs. **(B)** Addition of IL-6 rescued electrotaxis in cells that were transfected with shRNA against Cav-1. Scale bars = 50 μm. **(C)** migration directedness and speed of H1650-M3-plvt1351 cells in an EF of 100 mV/mm for 2 hrs, with/without IL-6 treatment. Data are from at least 100 cells from 3 independent experiments and shown as mean ± S.E.M. ^*^, p<0.01 and †, p<0.05, when compared with that of the control group. **(D)** Addition of IL-6 rescued activation of STAT3 in Cav-1 KD H1650-M3 cells. Western blotting analyzed the expression of indicated markers on protein extracts that were obtained from differentially treated cells, and β-actin was used as a loading control. Similar results were obtained in three independent experiments. See Supplementary Movie 5.

Next, we asked whether STAT3 was active downstream of Cav-1 in terms of modulating H1650-M3 electrotaxis. This was investigated by determining the effect of Cav-1 knock-down on EF-induced activation of STAT3. As expected, enhanced phosphorylation of STAT3 was observed in parental H1650-M3 cells and in those cells transfected with control shRNA (Figure 6A). Cav-1 knock-down resulted in decreased phosphorylation of STAT3, which was even further inhibited when an EF was applied. These results suggested that Cav-1 knock-down abolished EF-induced activation of STAT3 in H1650-M3 cells.

We next questioned whether induction of STAT3 activation in Cav-1 knock-down cells restored electrotaxis. The inflammatory cytokine IL-6, which is known to activate STAT3, was added to the culture medium of cells that had been transfected with shRNA against Cav-1. Western blot analysis confirmed STAT3 activation in an EF in the presence of IL-6 (Figure 6D). Treatment with IL-6 recovered galvanotaxis and cells migrated directionally to the cathode (Figure 6B and Supplementary Movie 5). Quantitative analyses of galvanotaxis migration showed complete recovery of the directedness values in these experiments (Figure 6C).

## DISCUSSION

Directed cell migration is a key step in tumor progression. Endogenous EFs have been shown to guide cancer cell migration, yet the underlying mechanism of the positive correlation between metastatic ability and electrotactic response is poorly understood. In this study, we demonstrated that: 1. outward currents were detected on the surface of a lung tumor with a vibrating probe; 2. highly-invasive H1650-M3 lung cancer cells migrated directionally toward the cathode in a voltage-dependent manner; 3. Cav-1-mediated STAT3 activation contributed to EF-guided migration.

### Endogenous EFs may be an important guidance cue for lung cancer cell migration

Endogenous EFs arise at sites of wounding of epithelial tissues, which was evidenced by detecting both current and field strength, based on different techniques. Using a vibrating probe, which is an ultrasensitive micro-probe that measures electric current non-invasively [24], we and others have measured outward currents of 4-10 μA cm^-2^ in corneal or skin wounds of experimental animal models in the rat, cow, and in human subjects [24, 28]. Meanwhile, an EF of 42-200 mV/mm was detected in skin wounds based on microelectrode techniques. The current study detected electric currents of 10-15 μA cm^-2^ around a tumor. Therefore, one could predict that the field strength used in the current study *in vitro* was comparable to the field strengths around a tumor *in vivo*. Endogenous EFs also exist between cancerous and normal tissues.

In breast cancer, electrical signals can be measured on the skin surface above the breast lesion and this non-invasive electropotential measurement has been used as a clinical method for breast cancer diagnosis and evaluating invasive potency [29–31]. Previously, several studies had demonstrated the directional movement of different types of cancer cells in response to dcEF [9, 11, 12, 32]. Further, in mammary and prostate ducts, the polarity of endogenous EFs correlated with the direction of their breast- or prostate-tumor cell migration in an EF *in vitro* [12, 29]. EFs were even suggested to be a powerful guidance signal that had the capacity to override other well-accepted cues, including mechanical forces, chemical signals, and contact inhibition [8]. In the current study, the direction of electric currents was towards the outer space of the tumor. *In vitro* results showed that human lung cancer H1650-M3 cells responded to EFs by migrating towards the cathode, which is in accordance with endogenous EF polarity. Collectively, observations support the hypothesis that endogenous EFs in the tumor microenvironment might serve as a guidance cue that directs lung cancer cell migration, thus promoting cancer invasion and metastasis.

### Cav-1 determines electrotaxis of lung cancer cells

Further investigation of signaling mechanisms of improved electrotaxis in highly-metastatic cancer cells will lead to an improved understanding of the electrical control of cancer cell migration. The striking difference in electrotaxis of H1650-M3 and H1650 cells is intriguing and may offer clues for possible mechanisms. In the current study, Cav-1, which is an integral membrane protein, was highly expressed in H1650-M3 cells. EF stimulation enhanced phosphorylation of Cav-1 in H1650-M3 cells, indicating that Cav-1 activation might play a role in cell electrotaxis. The essential role of Cav-1 in electrotaxis of H1650-M3 cells was further confirmed by shRNA KD of Cav-1, which abolished the electrotactic response of these cells. Previously, high expression of Cav-1 was demonstrated to be associated with enhanced malignancy, including multi-drug resistance and metastasis [33, 34]. In lung adenocarcinoma cells, Cav-1 is sufficient to promote filopodia formation, cell migration and increase metastatic potential [35]. Thus, our results, together with those findings, indicate that Cav-1 signaling mediates electrotaxis of lung cancer cells.

Precisely how Cav-1 senses an EF remains unknown. As discussed in a previously published review, ion channels and mechanosensitive channels might be potential candidates [11]. Fluxes of Ca^2+^, K^+^, Na^+^ and Cl^−^ were induced after wounding of the cornea, and increased transport of Cl^−^ forms a significant portion of the wound electrical current [28]. Blocking the voltage-gated Na^+^ channel (VGSC) significantly reduced the cathodal galvanotactic response of rat prostate cancer Mat-LyLu cells [12]. Application of voltage pulses across keratinocytes caused Ca^2+^ influx through voltage-gated Ca^2+^ channels (VGCCs) [36], while Ca^2+^ channel blockers reduced galvanotaxis [36, 37].

**Figure 7:**
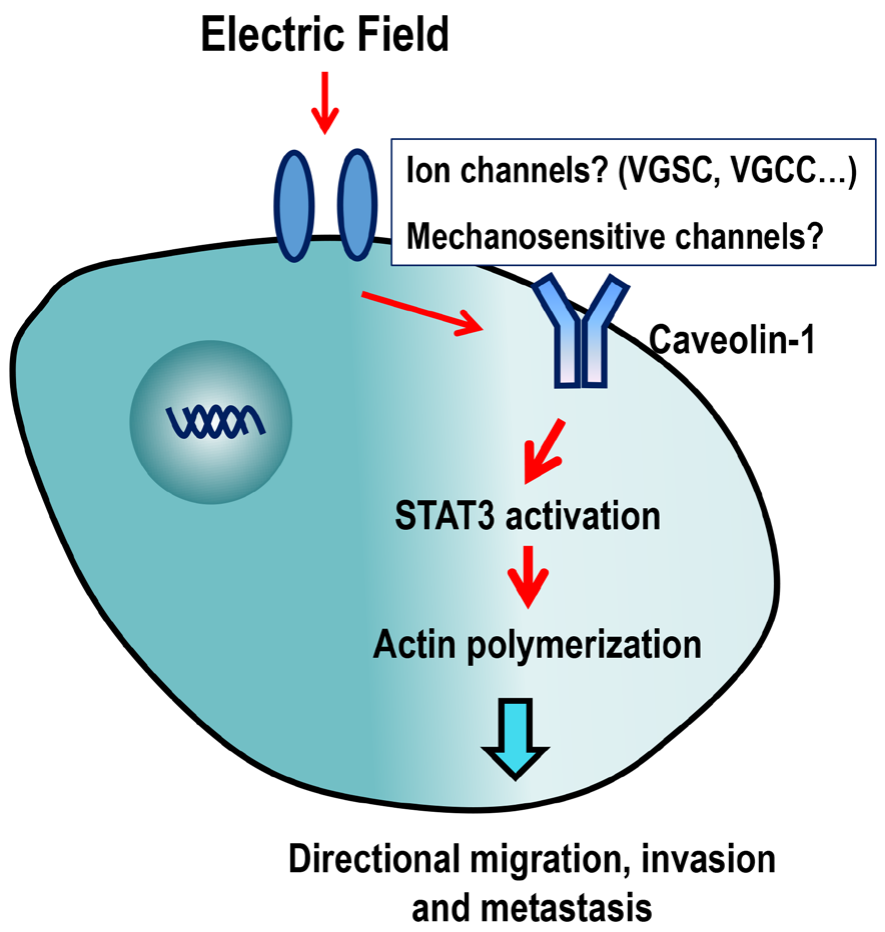
Schematic diagram showing the possible mechanisms of action of EF-guided lung cancer cell migration Caveolin-1 in cell membranes might sense extracellular EF stimulation through ion channels, following which, STAT3 is activated, which stimulates actin polymerization, directional migration, invasion and metastasis of cancer cells. VGSC: Voltage-gated Na^+^ channel; VGCC: voltage-gated Ca^2+^ channel.

Based on the important role of ion channels in cancer proliferation and metastasis, they may serve as novel viable targets for cancer therapy [38, 39]. Cav-1 activity can be modulated by ion channels. Chloride channel ClC-2 enhances intestinal epithelial tight junction barrier activity by regulating Cav-1 and caveolar trafficking of occludin [40]. In a rat brain glioma (C6) model, expression of Cav-1 protein at tumor sites was greatly increased after intracarotid infusion of minoxidil sulfate, which is a selective adenosine 5’-triphosphate-sensitive potassium channel (K (ATP) channel) activator [41]. These results, together with our findings, suggested that Cav-1 may be an important membrane sensor that transduces bio-electrical signals into cellular responses and promote cancer invasion and metastasis (Figure 7).

### STAT3: a possible key mediator in lung cancer electrotaxis and drug resistance

Understanding the downstream molecule responsible for Cav-1-mediated electrotaxis is important in the development of novel strategies to fight cancer invasion and metastasis. Cav-1 controls proliferation of metastatic lung cancer cells by regulating STAT3 signaling [20], while STAT3 can promote directional cell migration by organizing the actin cytoskeleton [42]. In the current study, we found that STAT3 was activated in H1650-M3 cells under EF stimulation, and inhibition of STAT3 activation significantly inhibited EF-guided cellular migration – an observation indicating that STAT3 is required for electrotaxis. Importantly, rescue of STAT3 activation in Cav-1 KD cells by adding IL-6 rescued electrotaxis of H1650-M3 cells, which suggested Cav-1 mediates electrotaxis by activating STAT3. Additionally, it should be noted that IL-6 may activate other signaling pathways other than STAT3.

Previously reported and yet controversial observation were reported regarding the relationship between Cav-1 and STAT3. Reduced levels of Cav-1 in human gastric adenocarcinoma cells resulted in significant STAT3 activation and enhanced cell proliferation [43]. Moreover, in breast cancer cells, activated STAT3 promoted invasion and brain metastasis by suppressing Cav-1 transcription by directly binding to its promoter [44]. These results suggest that different regulatory mechanisms may exist between Cav-1 and STAT3 activation in different types of cancer and in different stages of cancer progression. Thus, our results add to the notion that STAT3 activation by Cav-1 is required for lung cancer electrotaxis. Moreover, STAT3 plays an important role in drug resistance of non-small cell lung cancer (NSCLC). Activation of STAT3 induced cisplatin resistance in NSCLC by up-regulating anti-apoptotic and DNA repair-associated molecules [45]. We previously reported that in H1650-M3 cells, STAT3 activation determines EMT (epithelial-mesenchymal transition) and drug resistance to EGFR-TKI (epidermal growth factor receptor-tyrosine kinase inhibitor) [23]. Taken together, we propose that STAT3 may be an ideal candidate to target cancer invasion, metastasis and EGFR-TKI resistance.

### Limitations of the study

The current study has several limitations. First, the electrical signal *in vivo* was detected by the vibrating probe technique in the forms of μA cm^-2^, while the EF applied *in vitro* were in the form of mV/mm. Thus, we suggest that it is preferred to add detection of electrical signals using the microelectrode techniques, which can directly measure field strengths *in vivo*. Besides, it is also preferred to detect electrical signals from tumors generated from H1650 cells. Second, for mechanistic experiments, addition of more pairs of Cav-1 shRNA and STAT3 shRNA in H1650-M3 cells and over-expression of Cav-1 or STAT3 in H1650 cells would add increasing weight of evidence supportive of the importance of Cav-1 and STAT3 in electrotaxis. Additionally, application and use of a greater variety of cell-lines than those used in the current study would be preferred. Third, after Cav-1 KD or treatment with Stattic, cells showed changes in morphology and became clumped in appearance. Also their ability to migrate had also decreased. Thus, changes in cell morphology and migration might have influenced cellular electrotaxis.

In summary, endogenous EFs are present in the tumor microenvironment, and are likely to be involved in the pathophysiological process of cancer invasion. Precisely how highly-invasive lung cancer cells sense EFs is poorly understood. Herein, we detected electric currents on the surface of lung tumors. *In vitro*, highly-metastatic lung cancer H1650-M3 cells displayed electrotaxis in a voltage-dependent manner, which was controlled by Cav-1-mediated STAT3 activation. Collectively, endogenous EFs may represent an important guidance cue and stimulate directional migration of lung cancer cells, thus facilitating invasion and metastasis.

## MATERIALS AND METHODS

### Cell-lines and reagents

Erlotinib-sensitive H1650 cells and erlotinib-resistant H1650-M3 cells were kindly provided by Dr. Rafaela Sordella from Cold Spring Harbor Laboratory. Cells were cultured in RPMI 1640 medium (HyClone, Logan, UT, USA) with Earle's salts supplemented with 10% fetal bovine serum (FBS, Gibco, Waltham, MA, USA), 2 mM L-glutamine solution (Gibco), 100 U/ml penicillin (HyClone) and 100 μg/ml streptomycin (HyClone) at 37°C, with 5% CO_2_, and 90% humidity. Recombinant human IL-6 (rhIL-6) was purchased from PeproTech (Rocky Hill, NJ, USA). Stattic was obtained from Tocris Bioscience (Bristol, UK).

### Electrical field stimulation and drug treatment

Methods of applying EFs have been described previously [5, 25]. Briefly, cells were seeded into an electrotaxis chamber on a dish (Falcon tissue culture dishes, BD Biosciences, Franklin Lakes, NJ, USA) and left to adhere overnight in a 5% CO_2_ incubator. Then a No.1 coverglass was applied as a roof and sealed with high vacuum silicone grease (Dow Corning Corp., Midland, MI, USA) so that the final dimensions of the chamber were 24 mm × 8 mm × 0.2 mm. CO_2_ independent culture medium (Gibco) plus 10% FBS was used to maintain stable pH. Direct current was applied through agar-salt bridges connecting silver/silver chloride electrodes in Steinberg's solution to pooled medium on each side of the galvanotaxis chamber. Cells were exposed to 0-200 mV/mm steady EFs for the indicated periods of time. Time-lapse images were acquired using a Live Cell Station (Delta Vision, API, USA).

Stattic was used to attenuate STAT3 activation in H1650-M3 cells. Cells were treated with 0.5μM Stattic for 2 hours, then exposed to an EF of 100 mV/mm for 2 hours in the presence of the inhibitor. To activate STAT3 in Cav-1 KD H1650-M3 cells, IL-6 was added to the culture medium for 6 hours, then cells were subjected to EF stimulation in the continuous presence of IL-6.

### Quantitative analysis of cell migration

Cell migration was quantified using ImageJ software (NIH, Bethesda, MA, USA) with MTrackJ and Chemotaxis tool plugins as previously described [5]. Directedness (cos θ) was used to quantify how directionally the cells migrated, where θ is the angle between the field vector and the cell migration direction. The average directedness value of a population of cells reflected how directionally the cells had moved. Values close to 0 represented random cell movement, those close to 1 represented cells moving towards the cathode, and those close to -1 represented cells moving towards the anode. Cell migration speed was quantified as trajectory speed, which is the total length of the migration trajectory of a cell (Tt) divided by the given period of time.

### Western blot assay

Cells grown and treated as indicated were collected and total protein was extracted. The following primary antibodies were used: rabbit monoclonal anti-phosphorylated Caveolin-1, rabbit monoclonal anti-phosphorylated STAT3, rabbit monoclonal anti- Caveolin-1, rabbit monoclonal anti-STAT3 (all from Cell Signaling Technology, Danvers, MA, USA). Horseradish peroxidase-conjugated goat-anti-rabbit antibody (Thermo Scientific, Waltham, MA, USA) was used as a secondary antibody. The control for equal protein loading was assessed with an anti-β-actin antibody (Cell Signaling Technology).

### Immunohistochemistry

For immunofluorescence, cells were washed with PBS and fixed in 4% paraformaldehyde at room temperature for 30 min. Non-specific binding was blocked using 10% normal goat serum (Sigma). Cells were incubated with the following primary antibodies after being diluted in PBS with 1% bovine serum albumin at 4 °C overnight: rabbit monoclonal anti-phosphorylated Caveolin-1, rabbit monoclonal anti-phosphorylated STAT3, rabbit monoclonal anti- Caveolin-1 (Cell Signaling Technology). Then, cells were washed twice with PBS and incubated with secondary antibodies at 37 °C for 30 min as follows: FITC-conjugated goat-anti-rabbit IgG (Abcam, Cambridge, UK) or TRITC-conjugated goat-anti-rabbit IgG (Sigma, St. Louis, MO, USA). The slides were mounted in mounting medium with 4’, 6-diamidino-2-phenylindole (DAPI; Vector Laboratories, Burlingame, CA, USA) and viewed with a live cell station (Delta Vision, API).

### Transfection of shRNA

Synthetic, self-complementary oligonucleotides (59 nt) carrying shRNAs against the human mRNA of caveolin-1 (NM_001753) were designed with the following sequences:

5′-Ccgg-GACGTGGTCAAGATTGACTTT-CTCG AG-AAAGTCAATCTTGACCACGTC- TTTTTTg-3’ and

5′-aattcaaaaaa-GACGTGGTCAAGATTGACTTT-AAAGTCAATCTTGACCACGTC-3’.

The negative control oligonucleotides were likewise designed with the following sequences: 5′-Ccgg-TTCTCCGAACGTGTCA CGT-TTCAAGAGA-ACGTGACACGTTCGGAGAA- ttttttg-3’ and

5′-aattcaaaaaa-TTCTCCGAACGTGTCAC GT-TCTCTTGAA-ACGTGACACGTTCGGAGAA-3’.

The pMagic 7.1 lentiviral vector, which is driven by the RNA polymerase III specific promoter hU6, was used for shRNA expression and was constructed by Sesh-biotech (Shanghai, China). This lentiviral vector contains genes that code for green fluorescent protein and for a selective marker conferring resistance to the antibiotic puromycin. Lentiviruses were generated by co-transfection of pMagic 7.1-shRNA plasmids with the lentiviral plasmids pCD/NL-BH-DDD and pLTR-G into 293FT cells using Lipofectamine 2000 (Invitrogen) according to the manufacturer's protocol. Lentiviruses were collected in high serum-containing media at 48 and 72 h following transfection, which were then pooled and frozen at -80 °C for later use. H1650-M3 cells were transduced with lentiviruses by centrifugation at 1000g for 15 min at room temperature with high-titre virus and 8 Polybrene (hexadimethrine bromide, Sigma-Aldrich) followed by incubation with virus at 37°C for 4-6 h. ShRNA-transduced cells were selected with 1μg/ml puromycin for 72 h. Knock-down was assessed by Western blotting.

### Xenografts and measurement of electrical currents

Methods for xenograft implantation have been described previously [23]. All animal protocols were approved by the Ethics Committee of the Third Military Medical University. In brief, 2 × 10^6^ H1650-M3 cells were injected subcutaneously into the back next to the left forelimb of 6-week-old female BALB/cA-nu mice (Laboratory Animal Center of Third Military Medical University, Chongqing, China). Tumors with a size of ~100 mm^3^ developed around day-14. Then, the full-thickness dermal layer with the tumor was peeled off with surgical scissors after anesthetization of the animal and placed into a 100mm-dish with mouse ringer solution (154 mM NaCl, 5 mM KCl, 2 mM CaCl2, 1 mM MgCl2, 11 mM D- Glucose, 5 mM HEPES buffer, pH 7.3, all from Sangon, Shanghai, China) for further detection of electrical currents. Methods for detection of endogenous electric currents have been described previously [24, 46]. In brief, the premade electrodes were obtained from World Precision Instruments (Sarasota, FL, USA). The electrode was plated with platinum (platinum chloride plating solution: 0.01% w/v lead acetate plus 1% H_2_PtCl_6_ 6H_2_O in dH_2_O). Using the Scanning Vibrating Electrode Technique (SVET system, Applicable Electronics, New Haven, CT, USA), a current of 200 nA was applied for 5 min, which was increased to 500 nA for 2 min. The current was increased to 800 nA and applied in 0.5 s bursts until reaching the final tip size. The vibration of the electrode was adjusted through amplitude and frequency. Probes should be vibrated at an amplitude that approximates twice the tip diameter, and motion should be in a straight line, and should not exceed 20 degrees away from the true axis. Immediately before use, the probe was calibrated with a reference electrode in a dish (containing mouse ringer solution) to apply a current of exactly 60 nA. The probe was also calibrated at the end of the procedure in used solution to account for evaporation during the measurements. All procedures followed the SVET system manual. The full-thickness dermal layer with the tumor was fixed at the bottom of the Petri-dish with grease (High vacuum grease, Down Corning). The endogenous electrical currents were randomly measured three times at the tumor site. The normal skins beside the tumor site were measured as the control.

### Statistical analysis

All data are presented as mean ± standard error of the mean (SEM). Statistical analyses were measured by unpaired, two-tailed Student's t test and statistical significance was assumed at an alpha value of p < 0.05.

## SUPPLEMENTARY MATERIALS FIGURES


