# Correction: Caveolin-1-mediated STAT3 activation determines electrotaxis of human lung cancer cells

**DOI:** 10.18632/oncotarget.25675

**Published:** 2018-06-15

**Authors:** Li Li, Kejun Zhang, Conghua Lu, Qin Sun, Sanjun Zhao, Lin Jiao, Rui Han, Caiyu Lin, Jianxin Jiang, Min Zhao, Yong He

**Affiliations:** ^1^ Department of Respiratory Disease, Daping Hospital, Third Military Medical University, Chongqing 400042, China; ^2^ Department of Clinical Laboratory, Daping Hospital, Third Military Medical University, Chongqing 400042, China; ^3^ School of Life Sciences, Yunnan Normal University, Kunming 650500, China; ^4^ State Key Laboratory of Trauma, Burns and Combined Injury, Daping Hospital, Third Military Medical University, Chongqing 400042, China; ^5^ Department of Dermatology, Institute for Regenerative Cures, University of California, Davis, CA 95817, USA

**This article has been corrected:** The updated acknowledgements are given below:

**ACKNOWLEDGMENTS**

We thank Dr. Raffaella Sordella from Cold Spring Harbor Laboratory for kindly providing the valuable cell lines used in the current study. We thank Mrs. Juan Du (State key laboratory of Trauma, Bruns and combined injury, Third Military Medical University) for taking immunofluoresence images. The work in Zhao Lab was supported by AFOSR grant no. FA9550-16- 1-0052.

Original article: Oncotarget. 2017; 8:95741-95754. https://doi.org/10.18632/oncotarget.21306

