# Supplementary material for: Caveolin-1-mediated STAT3 activation determines electrotaxis of human lung cancer cells
Source: Oncotarget. 2017 Sep 28;8(56):95741–54. doi: 10.18632/oncotarget.21306 (PMC5707056; doi:10.18632/oncotarget.21306)
Supplement: Supplementary file 1 [file oncotarget-08-95741-s001.pdf]

Caveolin-1-mediated STAT3 activation determines electrotaxis of human lung cancer cells

SUPPLEMENTARY MATERIALS

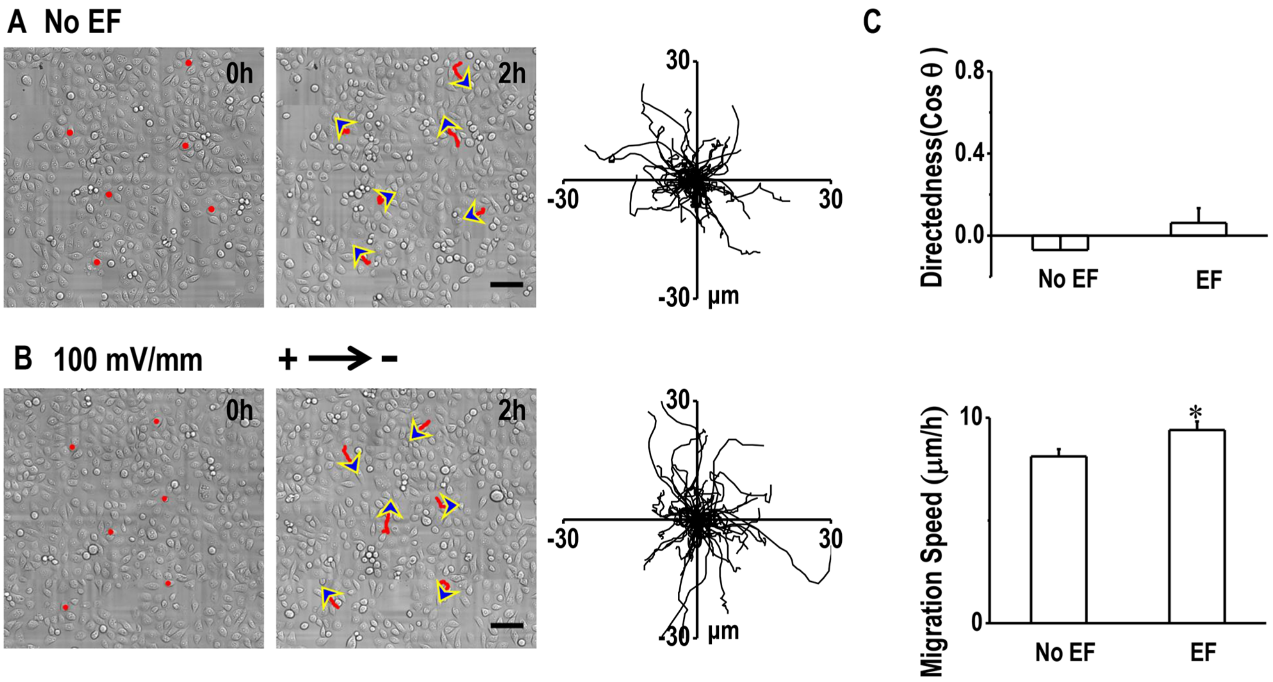

Supplementary Figure 1: H1650 cells migrate randomly in an EF. (A) Time lapse photographs of H1650 cells in the absence of EFs for 2 hours. Red lines and blue arrows represent migration paths and direction. Accumulated migration trajectories were presented with starting positions placed at the origin (0, 0). **(B)** H1650 cells migrated randomly in an EF of 100 mV/mm for 2 hours. **(C)** Migratory directedness and speed of H1650 cells under an EF of 100 mV/mm for 2 hours. The data are shown as Mean ± S.E.M. *, $p<0.05$ when compared with no EF control. See Movie 2.

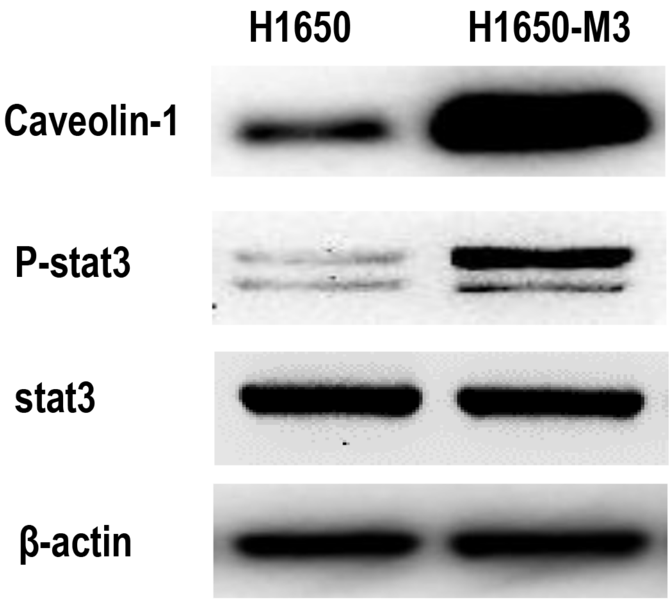

Supplementary Figure 2: Expression of Caveolin-1 and STAT3 in H1650 and H1650-M3 cells. Western Blot analysis was performed to detect the expression of Caveolin-1, phosphorylated STAT3 and total STAT3 in H1650 and H1650-M3 cells. Similar results were obtained in three independent experiments.

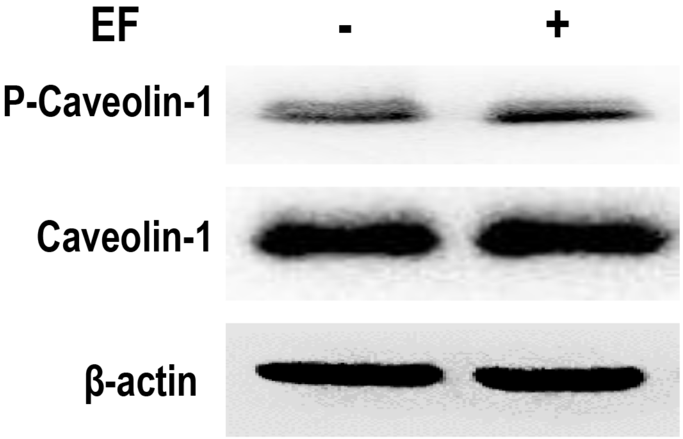

Supplementary Figure 3: Expression of phosphorylated and total Caveolin-1 in H1650 cells under EF stimulation. Western Blot analysis was performed to detect the expression of phosphorylated and total Caveolin-1 in H1650 cells with or without EF stimulation. Similar results were obtained in three independent experiments. EF = 100 mV/mm for 2 hours.

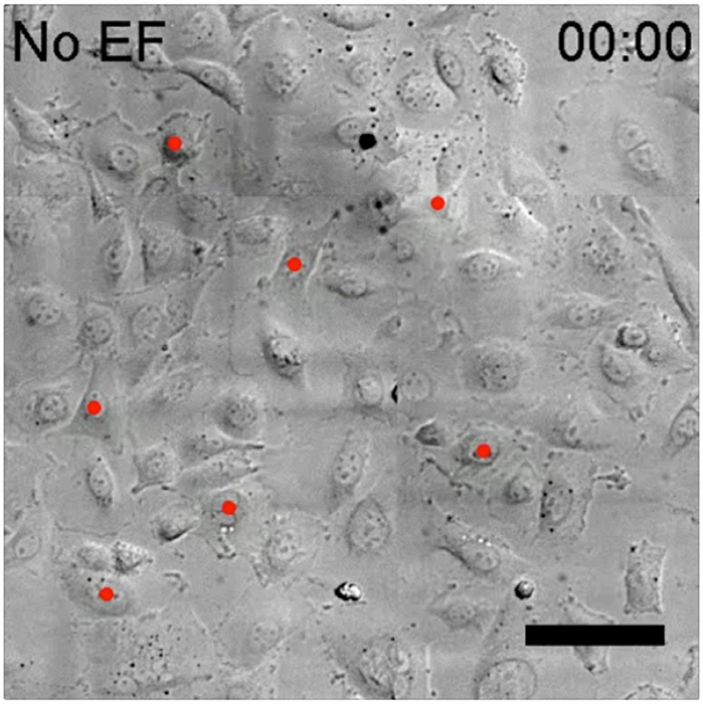

Supplementary Movie 1: H1650-M3 cells migrate to the cathode in an EF. Time is in hh:mm style.

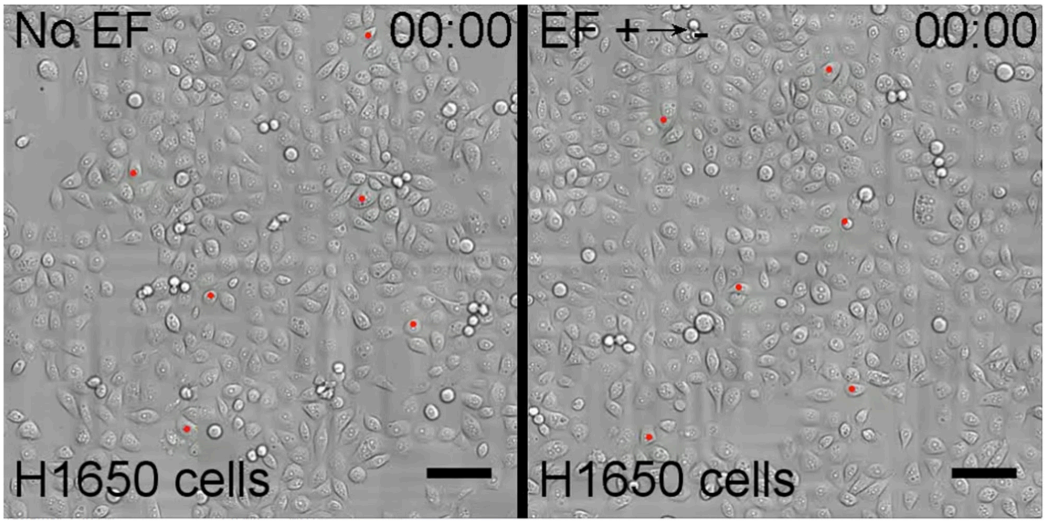

Supplementary Movie 2: H1650 cells migrate randomly in an EF. Time is in hh:mm style.

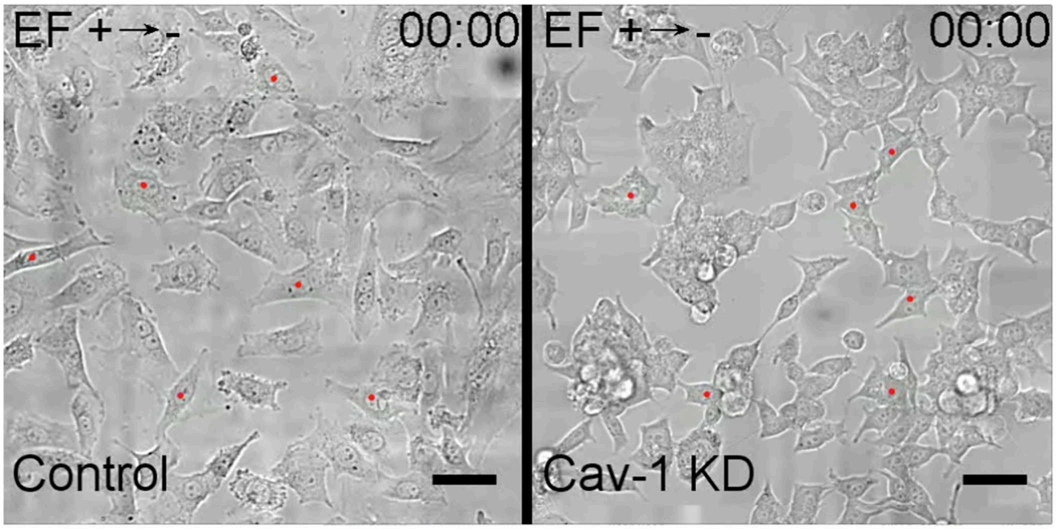

Supplementary Movie 3: shRNA knockdown of Cav-1 decreased directional migration of H1650-M3 cells in an EF. Time is in hh:mm style.

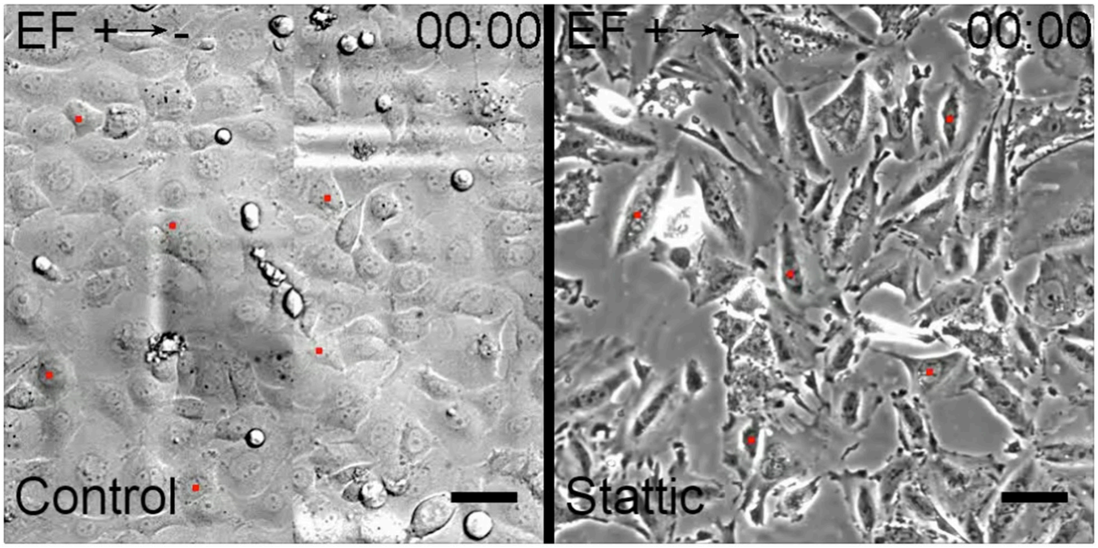

Supplementary Movie 4: Pharmacological inhibition of STAT3 abolished electrotaxis of H1650-M3 cells. Time is in hh:mm style.

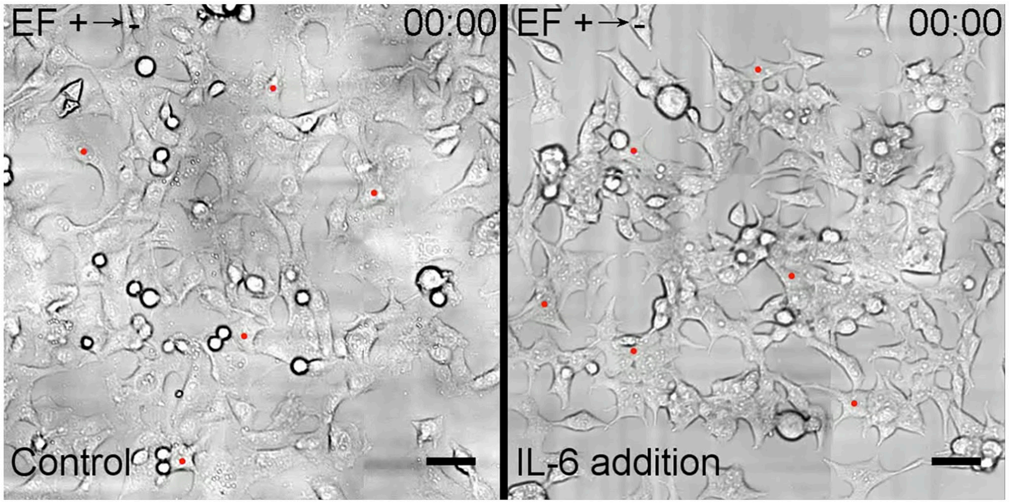

Supplementary Movie 5: Addition of IL-6 rescued electrotaxis in cells transfected with shRNA against Cav-1. Time is in hh:mm style.
